# Cucurbitacin B inhibits proliferation and induces apoptosis via STAT3 pathway inhibition in A549 lung cancer cells

**DOI:** 10.3892/mmr.2014.2581

**Published:** 2014-09-18

**Authors:** MENG ZHANG, ZHI-GANG BIAN, YI ZHANG, JIA-HE WANG, LIANG KAN, XIN WANG, HUI-YAN NIU, PING HE

**Affiliations:** 1 Department of Geriatrics, Shengjing Hospital of China Medical University, Shenyang, Liaoning 110004, P.R. China; 2 Department of Otolaryngology, Shengjing Hospital of China Medical University, Shenyang, Liaoning 110004, P.R. China

**Keywords:** A549 lung cancer cell line, cucurbitacin B, apoptosis, STAT3

## Abstract

Natural products are a great source of cancer chemotherapeutic agents. The present study was conducted to investigate whether cucurbitacin B (CuB), one of the most potent and widely used cucurbitacins, inhibits proliferation and induces apoptosis in the A549 lung cancer cell line. Furthermore, CuB induced apoptosis of A549 cells in a concentration-dependent manner, as determined by fluorescence microscopy, flow cytometry and transmission electron microscopy. The present study also demonstrated that CuB dose-dependently inhibited lung cancer cell proliferation, with cell cycle inhibition and cyclin B1 downregulation. Apoptosis induced by CuB was shown to be associated with cytochrome *c* release, B-cell lymphoma 2 downregulation and signal transducer and activator of transcription 3 pathway inhibition. CuB may prove to be a useful approach for the chemotherapy of lung cancer.

## Introduction

Lung cancer is one of the most common malignant tumor types worldwide, with increasing incidence and mortality rates (1). Despite the development of improved therapy modalities in the past two decades, the five-year survival rate has remained <15% (2). Several studies have suggested that conventional therapies may have reached a therapeutic plateau. Therefore, the current challenge is to investigate new therapeutic agents to treat lung cancer and other malignancies.

**Figure 1:**
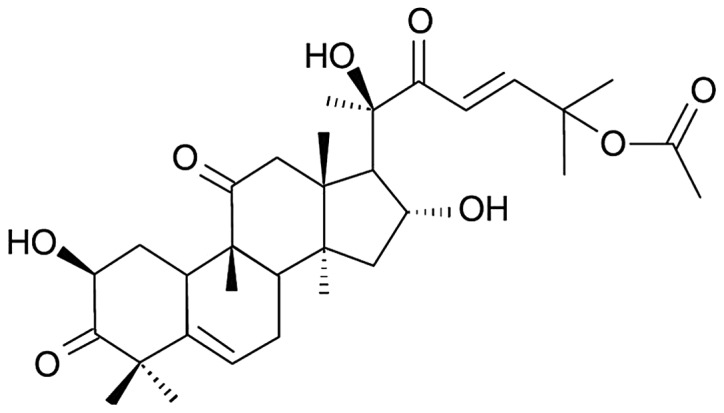
Chemical structure of cucurbitacin B.

Previously, there has been growing interest in the use of natural products as a new source of anticancer drugs (3,4). Cucurbitacins are compounds originally isolated from Cucurbitaceae plants (5). They are a group of diverse triterpenoid molecules with a number of biological properties, including cytotoxic, antitumor, hepatoprotective, anti-inflammatory, antimicrobial, antihelminthic and cardiovascular activities (6,7). Cucurbitacin B (CuB) (Fig. 1) is one of the most potent and widely used cucurbitacins (5). Accumulated evidence demonstrated that CuB induces apoptosis and inhibits the growth of various human cancer cell lines (5,7,8).

It was reported that numerous components of herbal medicine may induce apoptosis in lung cancer cells through the mitochondrial pathway (9,10). In another study, CuB treatment increased the protein levels of caspase-9 in the pancreatic cancer cell line Panc-1 (11). However, whether CuB is able to cause apoptosis of lung cancer cells in a mitochondria-dependent manner remains elusive.

In the present study, the anticancer effect of CuB on A549 lung cancer cells was investigated. The effect of CuB on cell proliferation, cell cycle distribution, apoptosis, caspase activity and cytochrome *c* release was examined. In addition, the possible mechanisms underlying this effect were investigated by screening a panel of proteins relevant to cell proliferation and apoptosis pathways.

## Materials and methods

### Reagents and chemicals

Highly purified CuB was purchased from the National Institute for the Control of Pharmaceutical and Biological Products (Beijing, China). RPMI-1640 and trypsin were purchased from Biological Industries (Kibutz Beit Haemek, Israel). Fetal bovine serum (FBS) and 3-(N-Morpholino)propanesulfonic acid (MOPS) buffer were purchased from Solarbio (Beijing Solarbio Science & Technology, Beijing, China). MTT, dimethyl sulfoxide (DMSO), propidium iodide (PI), Hoechst 33258 and rhodamine 123 were purchased from Sigma-Aldrich (St. Louis, MO, USA). Annexin V-fluorescein isothiocyanate (FITC) Apoptosis kit and bicinchoninic acid (BCA) protein assay kit were purchased from Key Gene (Nanjing, China). Mouse monoclonal antibodies specific to phosphorylated and total signal transducer and activator of transcription 3 (STAT3), cytochrome *c*, B-cell lymphoma 2 (Bcl-2), cyclin B1 and β-actin and the horseradish peroxidase (HRP) conjugated goat anti mouse immunoglobulin (Ig) G secondary antibody were purchased from Santa Cruz Biotechnology, Inc., (Santa Cruz, CA, USA). Enchanced Chemoluminescence Plus (ECL Plus) kit was purchased from Thermo Fisher Scientific, Inc., (Thermo Scientific Pierce, Waltham, MA, USA). All other chemicals were obtained from Sinopharm Chemical Reagent Shenyang Co., Ltd. (Shenyang, China).

### Cell culture

The human lung cancer cell line A549 was obtained from the China Center for Type Culture Collection (Wuhan, China). The cells were cultured in RPMI-1640 containing 10% fetal calf serum at 37°C in 5% CO_2_. The culture medium was replaced every day. The cells for the assays were detached using a solution of 0.25% trypsin and 0.02% EDTA.

### Cell viability assay

Viability was assessed by the MTT assay. Approximately 5×10^3^ cells were plated per well in 96-well plates and treated with different concentrations of CuB (0.02, 0.1, 0.5, 2.5, 12.5 and 62.5 μmol/l) for 24, 48 and 72 h, respectively. Corresponding DMSO and culture medium were used either as a control or empty control. For quantitation of cell viability, 25 μl MTT solution [(2 mg/ml in phosphate-buffered saline (PBS)] was added to each well, and the plates were incubated for an additional 4 h at 37°C. The medium was then removed and 150 μl DMSO was added to each well to solubilize the formazan crystals formed in viable cells. Each solution was measured spectrophotometrically at 570 nm (OD570) using an ELISA plate reader (Model 550; Bio-Rad, Hercules, CA, USA). At least three independent experiments were performed.

### Flow cytometry for cell cycle analysis

The cells were seeded into six-well plates at a concentration of 5×10^5^/well and allowed to attach in culture overnight, then treated with either 0.1 or 1 μmol/l CuB for 24 h and harvested. For cell cycle analysis, the cells were fixed in 70% ice cold ethanol at 4°C overnight. The cells were then washed with PBS, treated with RNase and stained with PI (100 μg/ml) in the dark for 30 min at room temperature. The samples were analyzed by a FACScan flow cytometer (BD Biosciences, Franklin Lakes, NJ, USA). At least three independent experiments were performed.

### Flow cytometric analysis of apoptosis

For Annexin V/PI apoptosis analysis, 5×10^5^ cells were plated per well in six-well plates and treated with either 0.1 or 1 μmol/l CuB for 24 h. Following harvesting, the cells were resuspended in cold PBS and stained using an Annexin V-FITC Apoptosis kit according to the manufacturer’s instructions. The cells were analyzed using a FACScan flow cytometer. At least three independent experiments were performed.

### Fluorescence microscopy

The cells (5×10^5^) were grown on coverslips placed into six-well plates. Following treatment with different concentrations of CuB (0.1 μmol/l, 1 μmol/l) or an equal concentration of DMSO for 24 h, the cells were washed twice with cold PBS, fixed with cold methanol and acetic acid (3/1, v/v) at 4°C overnight and stained with Hoechst 33258 for 30 min in the dark, washed again in PBS and finally mounted in mounting medium (80% glycerol in PBS). Morphological changes were analyzed under an E800 fluorescence microscope (Nikon, Tokyo, Japan).

### Transmission electron microscopy

The cells treated with 1 μmol/l CuB for 24 h were harvested and fixed with 3% glutaraldehyde overnight. Following removal of the primary fixative, the cells were washed three times with MOPS buffer, post-fixed in 1% osmium tetroxide (OsO_4_), dehydrated in an ethanol series and embedded in epoxy resin. Ultra-thin sections were double-stained with lead citrate/uranyl acetate prior to examination using a JEM-100CX transmission electron microscope (Japan Electron Optics Laboratory Co., Ltd, Tokyo, Japan).

### Caspase-3 and caspase-9 activity assay

The activity of caspase-3 and caspase-9 was detected by chromogenic substrate assay. The cells were seeded in 75 cm^2^-culture flasks and cultured for 24 h. The cells were then treated with 1 μmol/l CuB for 24 h and harvested. An equal volume of culture medium was added to the control group. The protein was then extracted with lysis buffer and quantified with a BCA protein assay kit. A total of 200 μg protein was prepared, diluted to a volume of 50 μl, 50 μl of 2× reaction buffer was added with 5 μl substrate of caspase-3 or caspase-9. A total of 50 μl lysis buffer and 50 μl 2× reaction buffer was added to the control group. Following incubation for 4 h at 37°C, the absorbance was measured at 405 nm. At least three independent experiments were performed.

### Detection of the mitochondrial membrane potential (Δψm)

The A549 cells (5×10^5^/well) were seeded into six-well plates and then treated with 0.1 μmol/l or 1 μmol/l CuB for 24 h. An equal amount of culture medium was added as the control. The cells were harvested and incubated with 10 mg/ml rhodamine 123 for 30 min at 37°C. The cells were resuspended with PBS and analyzed using a FACScan flow cytometer. At least three independent experiments were performed.

### Western blot analysis

The cells were seeded in culture flasks and then incubated with 0.1 μmol/l or 1 μmol/l CuB for 24 h. An equal amount of RPMI-1640 was added to the control group. Total extracted protein, cytoplasmic protein or mitochondrial protein were analyzed separately. The proteins were separated by SDS-PAGE and transferred to polyvinylidene fluoride membranes. The membranes were blocked with 5% non-fat milk and incubated overnight at 4°C with antibodies against phosphorylated and total STAT3, cytochrome *c*, Bcl-2, cyclin B1 and β-actin (1:1,000). Following incubation with peroxidase-conjugated anti-mouse IgG (1:10,000) at room temperature for 1 h, the proteins were visualized using an ECL Plus kit and detected using a ChemiDoc-It BioImaging system (UVP Inc., Upland, CA, USA).

### Statistical analysis

Values are expressed as the mean ± standard deviation. The statistical correlation of data was examined for significance by analysis of variance and Student’s t-test. P<0.05 was considered to indicate a statistically significant difference. These analyses were performed using SPSS 13.0 software (SPSS, Inc., Chicago, IL, USA).

## Results

### CuB inhibits A549 cell proliferation

**Figure 2:**
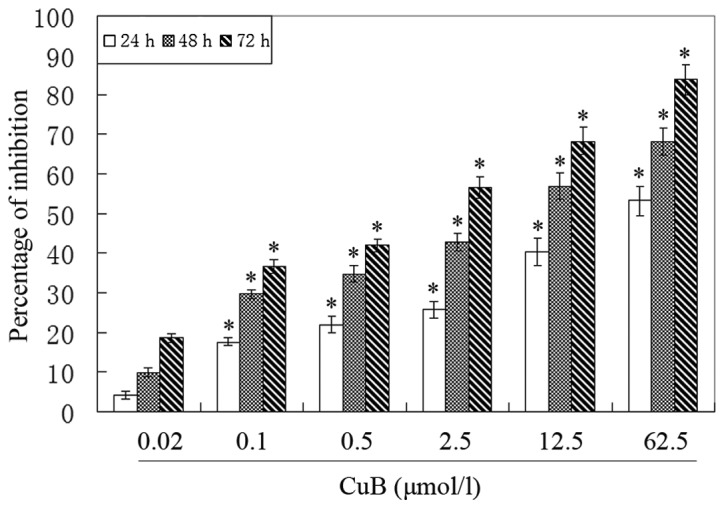
The proliferative inhibition effects of CuB on human lung cancer A549 cells. ^*^P<0.05 vs. the control group. CuB, cucurbitacin B.

To evaluate the effect of CuB on the proliferation of A549 cells, an MTT assay was performed. It was observed that the growth of A549 cells was suppressed in a dose- and time-dependent manner (Fig. 2).

### CuB induces G2/M cell cycle arrest in A549 cells

**Figure 3:**
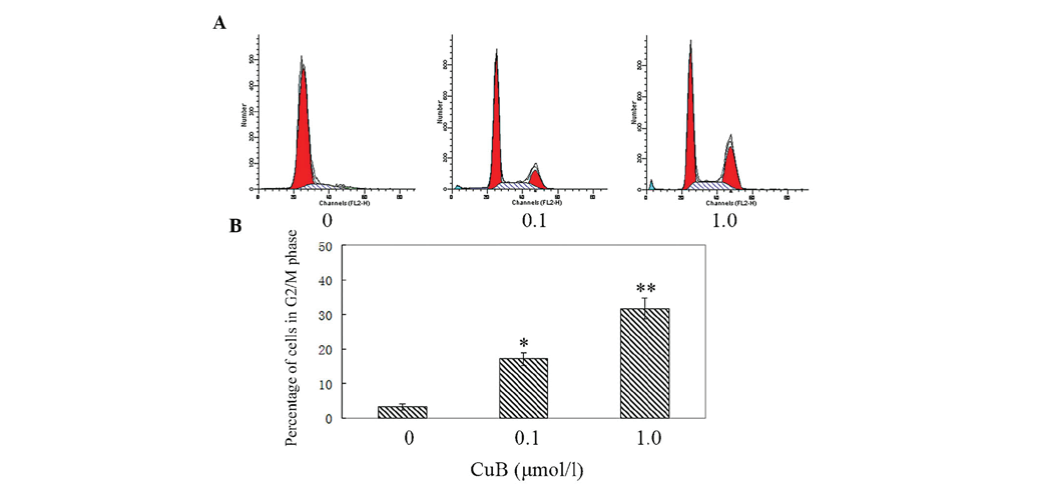
Flow cytometric cell cycle analysis. (A) A549 cells were treated with CuB (0, 0.1 and 1.0 μmol/l) for 24 h. The cells were then harvested and stained with propidium iodide. The cell cycle distribution was analyzed by flow cytometry. (B) Percentage of cells in G2/M phase in histograms. ^*^P<0.05 vs. the control group; ^**^P<0.01 vs. the control group. CuB, cucurbitacin B.

To further investigate the inhibitory effect of CuB on cell growth, the cell cycle distribution in A549 cells was examined by flow cytometry. The cells in the experimental groups were treated with 0.1 μmol/l and 1 μmol/l CuB. As shown in Fig. 3, when the dose of CuB was increased, the percentage of cells in the G2/M phase increased markedly. In the control group, the percentage of cells in G2/M phase was 3.26±1.02% and in the CuB high-dose group, the G2/M percentage was 31.78±3.69%. The results demonstrated that CuB induced G2/M arrest significantly.

### CuB induces apoptosis of A549 cells

**Figure 4:**
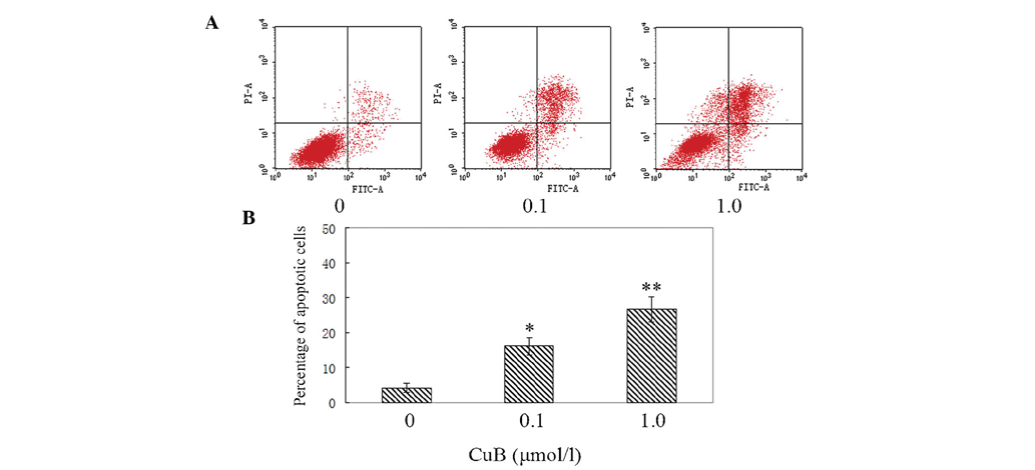
Flow cytometric quantification of apoptotic cells. (A) CuB-induced apoptosis in A549 cells as assayed by Annexin V/PI staining. A549 cells were treated with CuB (0, 0.1 and 1.0 μmol/l) for 24 h. The cells were then harvested and stained with Annexin V/PI prior to flow cytometric analysis of apoptosis. (B) Quantification of apoptotic cells from A in a bar chart. ^*^P<0.05 vs. the control group; ^**^P<0.01 vs. the control group. CuB, cucurbitacin B; PI, propium iodide; FITC, fluorescein isothiocyanate.

AnnexinV/PI analysis was employed to examine the effect of CuB on apoptosis in A549 cells. It was identified that CuB induced apoptosis of A549 cells evidently. The percentages of early and late apoptotic cells were significantly increased compared with the control group. The proportion of apoptotic cells in the treated cells was increased in a dose-dependent manner (Fig. 4).

**Figure 5:**
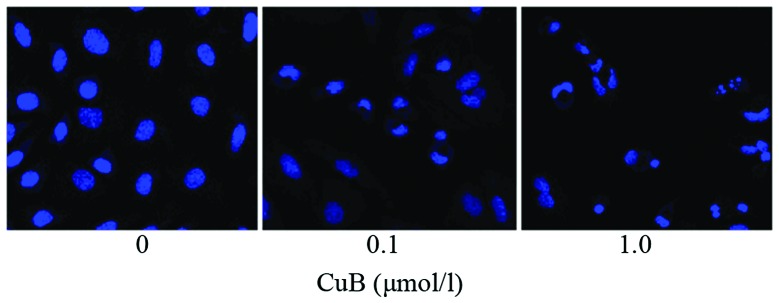
Cell apoptosis observed by Hoechst 33258 staining. A549 cells treated with CuB (0, 0.1 and 1.0 μmol/l) for 24 h. Apoptotic cells exhibited chromatin condensation and nuclear fragmentation. CuB, cucurbitacin B. Magnification, ×400.

Following treatment with CuB for 24 h, the A549 cells exhibited typical morphological hallmarks of apoptosis, including condensation of chromatin, karyopyknosis and nuclear fragmentation, which was observed using Hoechst 33258 staining (Fig. 5). As the dose of CuB increased, the rate of cell apoptosis increased.

**Figure 6:**
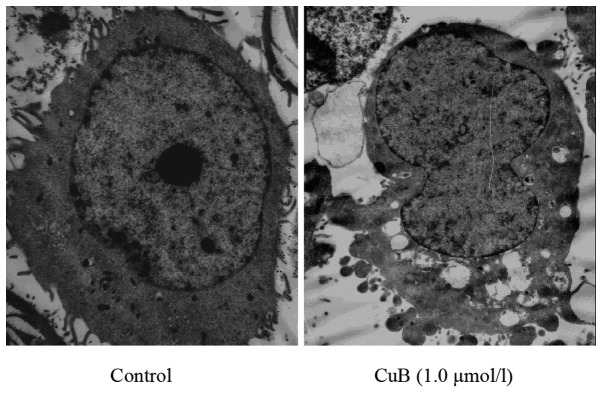
Transmission electron micrographs of A549 control cells and cells treated with CuB (1.0 μmol/l) for 24 h. The treated cells lost their pseudopodia and exhibited evident cell shrinking, intracytoplasmic vacuoles, chromatin condensation and mitochondria swelling (magnification, ×5,000). CuB, cucurbitacin B.

Ultrastructural changes caused by CuB in A549 cells were also examined. Transmission electron microscopic analysis demonstrated morphological alterations in the A549 cells treated with CuB. The cells treated with 1.0 μmol/l CuB for 24 h lost their pseudopodia and exhibited evidence of cell shrinking, intracytoplasmic vacuoles, chromatin condensation and mitochondrial swelling (Fig. 6).

### CuB induces caspase-3 and -9 activation

**Figure 7:**
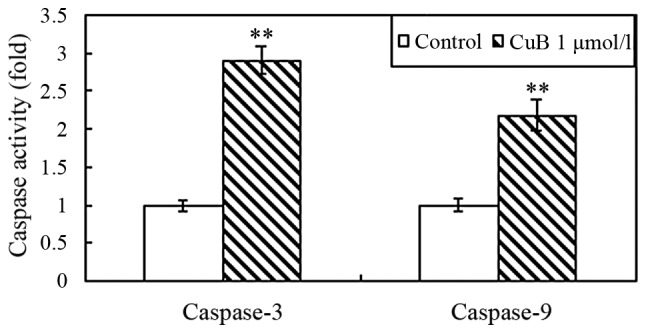
Effect of CuB on the activity of caspase-3 and -9 in CuB-induced apoptosis. A549 cells were treated with CuB (1.0 μmol/l) for 24 h. ^**^P<0.01 vs. the control group. CuB, cucurbitacin B; PI, propium iodide.

To further investigate the effect of CuB on apoptosis, the activation of caspase-3 and caspase-9 in A549 cells was examined using caspase activity assay kits. The proteolytic activity of both caspase-3 and caspase-9 was significantly increased following treatment with CuB for 24 h (Fig. 7).

### CuB induces disruption of the Δψm and cytochrome c release

**Figure 8:**
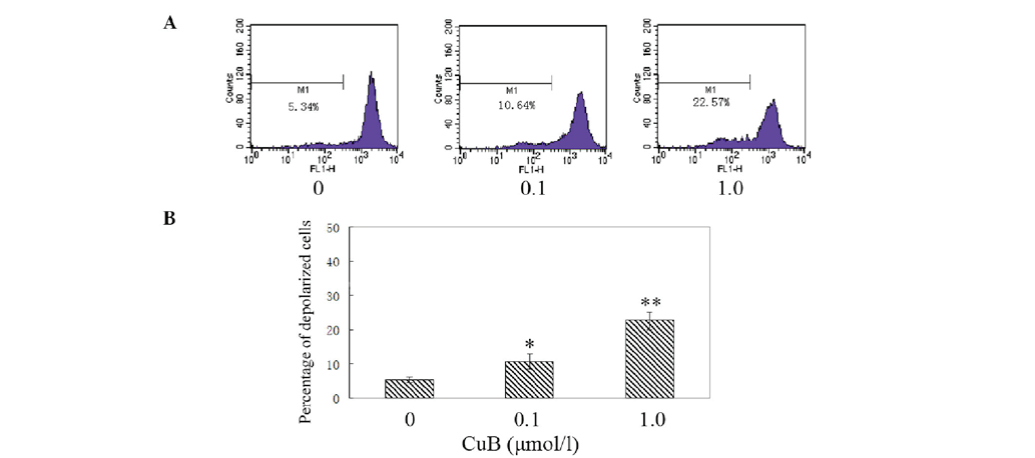
CuB induces disruption of ΔΨm. (A) A549 cells were treated with CuB (0, 0.1 and 1.0 μmol/l) for 24 h. The cells were then harvested, stained with rhodamine 123 and flow cytometric analysis was performed to analyze ΔΨm. (B) Quantification of the depolarization data from the histograms in A. ^*^P<0.05 vs. the control group; ^**^P<0.01 vs. the control group. CuB, cucurbitacin B; ΔΨm, mitochondrial membrane potential.

**Figure 9:**
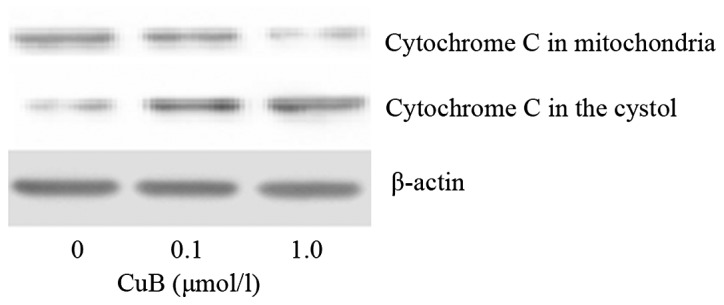
CuB induces the release of mitochondrial cytochrome C. A549 cells were treated with CuB (0, 0.1 and 1.0 μmol/l) for 24 h. Following isolation of the mitochondrial and cytosolic fractions, mitochondrial cytochrome C release was detected by western blot analysis. CuB, cucurbitacin B.

The effect of CuB on Δψm following CuB treatment was examined by rhodamine 123 staining and flow cytometric analysis. The integral under the Δψm curve decreased and shifted toward the left; therefore, the proportion of depolarized cells increased in a dose-dependent manner following CuB treatment (Fig. 8). The results indicated that cucurbitacin B treatment induced significant disruption of the Δψm. As cytochrome *c* release may be a limiting factor in caspase-9 activation and represents a control coordinating step in apoptosis, the ability of CuB to trigger cytochrome *c* release was examined in A549 cells. As demonstrated in Fig. 9, CuB treatment induced the release of mitochondrial cytochrome *c* into the cytosol.

### CuB downregulates the protein expression of phosphorylated (p)-STAT3, cyclinB1 and Bcl-2

**Figure 10:**
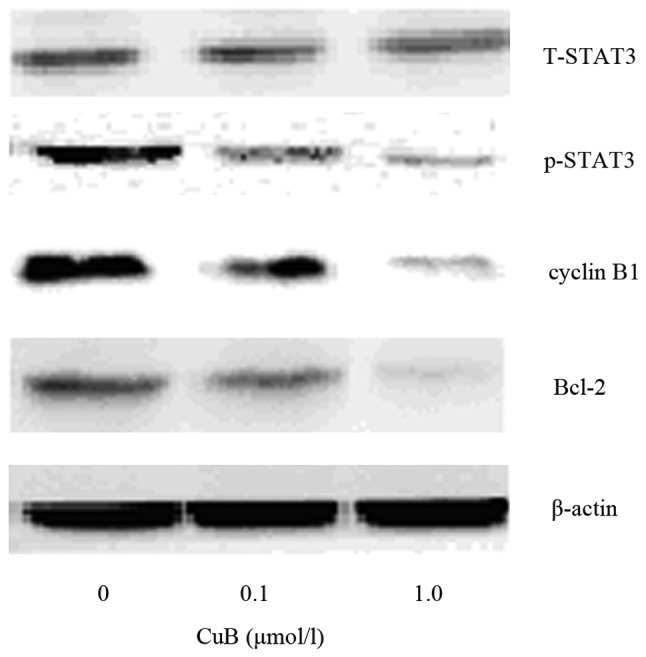
Effect of CuB on the expression of cyclin B1, p-STAT3, T-STAT3 and Bcl-2 by western blot analysis. A549 cells were treated with CuB (0, 0.1 and 1.0 μmol/l) for 24 h. The proteins were extracted, then cyclin B1, p-STAT3, T-STAT3, Bcl-2 and β-actin expression were analyzed by western blot. CuB, cucurbitacin B; P/T-STAT3, phosphorylated/total signal transducer and activator of transcription 3; Bcl-2, B-cell lymphoma 2.

To further examine the mechanisms of the effect of CuB on proliferation and apoptosis in A549 cells, a panel of proteins which are closely associated with cell growth and apoptosis were detected. CuB suppressed p-STAT3 in a dose-dependent manner, while it had no effect on the levels of total STAT3. Furthermore, it was identified that CuB treatment decreased the protein levels of cyclinB1 and Bcl-2 as well, which are downstream targets of STAT3 and are associated with cell growth and apoptosis. The results indicated that CuB affects proliferation and apoptosis through inhibiting STAT3 activation and subsequently decreased the levels of cyclin B1 and Bcl-2 protein expression (Fig. 10).

## Discussion

Cucurbitacin B is a compound originally isolated from Cucurbitaceae plants and has hepatoprotective biological properties. Accumulating evidence has indicated that CuB inhibits proliferation and induces apoptosis in several human cancer cell lines (5,11–13). In the present study, it was identified that CuB may induce apoptosis in the lung cancer cell line A549. In addition, CuB inhibited the proliferation rate of A549 cells in a dose- and time-dependent manner. Further study revealed that CuB treatment caused G2/M cell cycle arrest, elevated caspase-3 and caspase-9 activity, ΔΨm disruption and cytochrome *c* release. Examination of potential target protein expression revealed that CuB inhibited STAT3 phosphorylation, and downregulated cyclin B1 and Bcl-2 expression.

The induction of cell cycle arrest and apoptosis are common mechanisms proposed for the cytotoxic effects of anticancer drugs extracted from medicinal plants (14). In the present study the potential mechanism by which CuB inhibits cell proliferation was examined. Flow cytometry results demonstrated that CuB arrested cell cycle progression at the G2/M check point with a decreased G0/G1 ratio, thus inhibiting the cell proliferation rate. Accordingly, the expression of cyclin B1 was also decreased. Cyclin B1 is a regulatory protein involved in mitosis and may form a complex with cyclin-dependent kinase 1 (cdk1) (15). Cyclin B1-Cdk1 is involved in the early events of mitosis, including chromosome condensation, nuclear envelope breakdown and spindle pole assembly. Previous reports demonstrated that CuB was able to inhibit G2/M transition in breast cancer cells, laryngeal cancer cells and colon adenocarcinoma cells, which was in accordance with the results of the present study (12,16,17).

CuB was reported to induce apoptosis in various cancer cell lines, including laryngeal, pancreatic, colon and hepatocellular carcinoma (5,11,13,16). Using flow cytometry, fluorescence microscopy and transmission electron microscopy, it was demonstrated that CuB treatment induced lung cancer cell apoptosis in a dose-dependent manner. Mitochondrial dysfunction has been demonstrated to participate in the induction of apoptosis and has been suggested to be central to the apoptotic pathway. Further analysis of mitochondrial function revealed a disruption of ΔΨm and cytochrome *c* release. In addition, the levels of caspase-3 and caspase-9 activity were also upregulated, coupled with downregulation of the anti-apoptotic Bcl-2 protein. The Bcl-2 protein regulates apoptosis by controlling mitochondrial permeability (18). Bcl-2 resides in the outer mitochondrial wall and inhibits cytochrome *c* release (19). Upon release from the mitochondria, cytochrome *c* binds to apoptotic protease activating factor 1 and forms an activation complex with caspase-9 (20,21). Caspase-9 activates caspase-3 by proteolytic cleavage and caspase-3 then cleaves vital cellular proteins or other caspases (22). Based on the present results, CuB may activate the apoptosis cascade through initiating cytochrome *c* release and downregulating Bcl-2 protein expression.

Previous studies demonstrated that CuB suppressed the activation of STAT3, regulated STAT3 downstream genes and consequently inhibited tumor growth in several types of cancer (11,13). Accordingly, a decrease of STAT3 phosphorylation following CuB treatment was identified. The STAT family proteins have been demonstrated to have important roles in tumorigenesis (23–25). Recent *in vitro* and *in vivo* studies have revealed that several strategies to target STAT3 signaling have been proposed as cancer therapies (26,27). In addition, Bcl-2 was a downstream target gene of STAT3 signaling. The results indicated that CuB may downregulate Bcl-2 and induce apoptosis through inhibition of the STAT3 pathway.

In conclusion, the present study revealed that CuB inhibited proliferation in lung cancer cells, with cell cycle inhibition and cyclin B1 downregulation. CuB also induced lung cancer cell apoptosis through cytochrome *c* release, Bcl-2 downregulation and STAT3 pathway inhibition. CuB may serve as a potentially useful therapeutic strategy for patients with lung cancer.
